# The Principles of Medical Practice, Formerly and Now

**Published:** 1878-09

**Authors:** J. Dickson Smith

**Affiliations:** Atlanta, Ga.


### THE PRINCIPLES OF MEDICAL PRACTICE, FORMERLY AND NOW.

BY J. DICKSON SMITH, M.D., Atlanta, Ga.

At a point in the past history of medicine, and within the recollection of many practitioners of this day, its practice was dictated by theoretical and absurdly erroneous notions. The profession regarded diseases as real entities, possessing passions and volitious intentions inimical to human life; and by virtue of such malignant designs, diseases were considered the implacable foes of the human race. Such ontological view suggested imaginary strife for life and death, and placed the physician in the attitude of direct antagonism to diseases.

Hence the heroic practice in vogue at and anterior to this period already indicated. Looking upon disease as an extraneous foe that tended to pursue its ravages to the destruction of the life of the patient, the physician, assuming the aggressive, attacked it with violent measures, regardless of the damaging effects of his blows. He essayed to cure the patient by fighting and subduing his disease.

The physician, as well as the surgeon, cultivated the trait of boldness and daring, and aspired to reputation conferred by brilliant exploits and Waterloo operations. *Inflammation* was the disease, and *antiphlogistics* the remedies. The theory at that time entertained as to the nature and cause of inflammation brought into requisition a class of depletive and debilitating remedies, which tended to prostrate the patient and exhaust the vital forces. These were represented by blood-letting, cathartics, emetics, blisters, mercurialization, etc., and which were indiscriminately employed by authority of the theory and practice of the day for the arrest or subduction of the inflammation. Ignorant of the laws of pathology, the physician treated diseases under the general idea that they tend naturally to an unfavorable termination, and to prove fatal to the life of the patient unless cured by remedies. Hence they employed powerful remedies, and pushed them to an enormous extent, conjoined with a rigid system of dieting amounting almost to starvation. Most of the remedies employed illustrated the same aggressive spirit of attack upon the disease; and unmindful of the damaging effects of such blows upon the physical strength and vital powers of the patient, the practice was to invade the organism in search of the disease, for its arrest and subjugation.

Such epitome will suffice to indicate medical practice of twenty-five years ago, and anterior to that time. Without attempting to trace the history of medicine through succeeding years, we will notice some of the characteristic features of the sentiment and practice of our own day. Such comparison will develop a very striking contrast—indicating unmistakable advances in medical knowledge, and wonderful improvement in the management of most diseases. The practice has *materially* and *essentially* changed, and been placed upon a *distinctly different* basis. Actual knowledge acquired in the several departments of medical science has measurably unfettered the mind of the profession from the manacles of theory, system and traditional authority, and allowed sound sense and reason to exercise their rightful prerogative in dictating therapeutical indications in strict accordance with nature's laws. The great light that now guides the medical mind, and directs its practice in the management of the sick has been but recently developed from the study of the natural history of diseases. This light brings to view, for our guidance in therapeutics, the grand *conservative principle* of nature, as exemplified in all her laws, and which, in relation to our subject, is so forcibly exemplified in the anatomical conformation, and in the physiological and pathological processes of the human system as to give evidence of its Divine appointment.

The practical recognition of this conservative principle, together with a series of chemical facts directly to be mentioned, constitute the source and basis of the distinguishing features of present medical practice as compared with the past. Upon these are based all rational therapeutical indications for the management of diseases. Of the laws of pathology enough is already known to establish the following facts: Most diseases possess an intrinsic tendency to pursue a definite career, and end in accordance with its law of self-limitation; most diseases tend to run a favorable course and end in recovery; a very large proportion of the diseases ending fatally reach that result by *asthenia* or *exhaustion*, and very few by *apnœa.* Upon these facts from the laws of disease are based modern conservative therapeutics, and which distinguishes medical practice of to-day from the spoliative routine of twenty-five years ago.

The progressive practitioner of our day, instead of making war upon disease by violent means, quietly allies himself with nature for her protection and defense, and to guard her safely against the inroads and damaging effects of existing disease. Restricting himself simply to his legitimate office of *minister*, and not *master*, of nature, as formerly arrogated, the physician seeks to suppoort nature in her faltering weakness, and, by safe therapeutic and hygienic measures, to maintain in the organism that degree of strength and vigor that will enable it to tolerate the disease till it has passed through its natural career. True, much may be done by way of mitigating violence of symptoms and palliating the painful effects of disease; yet the main power of the physician for good will be most manifest by such alliance with nature in her efforts to guard the organism, hold in abeyance the spoliative effects of its malady, and counteracting its every tendency towards dissolution. In short, the legitimate office of the physician is rather to treat and care for the patient, than fight the disease that is quietly running its allotted course. It is also his province to secure the sick against the damaging effects of any injurious medication or meddlesome interference. Hence the measurable abandonment of that class of potential measures formerly employed as antiphlogistics. They interrupt the conservative designs of nature, fail to arrest or cure the inflammation, and are directly perturbatory, spoliative, and absolutely exhausting to the physical powers and strength of the patient. And, moreover, clinical experience renders it exceedingly problematical as to whether this class of remedies exert any material effect upon the intensity of inflammation, its results, or its duration; and consequently their depreciation in medical confidence, and comparative disuse in practice for such special object.

It is not to be denied, however, that the profession is possessed of very many valuable therapeutic agencies known to possess special alleviating and specific curative properties. But it must be admitted that the class of *specifics* and *curative* remedies is comparatively small—the main catalogue acting only indirectly as medicinal agents when applied in accordance with general therapeutic principles.

Medical practice, under the advanced and progressive sentiment of the present day, comprises everything calculated to prevent impairment of, or that tends to develop and sustain, the powers of life. Medication is withheld, or simple palliatives used, or active remedies employed with great circumspection, according to the case and the intrinsic tendency of the disease towards favorable or fatal termination. Abjuring officious interference, it prescribes active and potential therapeutic measures only with a view to well-defined objects, and in accordance with rational principles. It does not seek to control diseases by measures which, if not successful, may diminish the chances of the patient passing safely through the several stages of the disease; and in cases known to be beyond the power of medicine to arrest or abridge, it seeks merely to modify and palliate the symptoms as they arise, and to keep up the patient with sustaining treatment. It reluctantly interferes actively with the course of diseases known to have a self-limited tendency. Instead of random shots at the disease with formularized prescriptions of potent drugs and nauseous draughts, we prefer to assist nature, by supporting measures, in enduring, and the vital forces in triumphing over, the invading foe.

Tonic medicines, alcoholic stimulants, nutritious diet, and the whole regime of hygienic influences, as supporting measures, constitute the important weapons of warfare to be wielded by the physician in the "struggle of life and death" between disease and the life of its subject. These sustaining measures tend to promote the conservative efforts of nature in placing and maintaining the system in the best possible condition for tolerance of the disease. Both common sense and experience endorse the plain proposition that, the nearer the condition of the general health is maintained towards its normal standard, the greater the chances for recovery from disease, and the better fortified is the system for enduring its continuance. Not many diseases prove fatal *per se*—death being generally caused, not by irremediable injury to the part affected, but by occurring complications, and the general disorder and failure of the vital powers. Hence the indication to endeavor to obviate the general disorder, prevent failure of the vital forces, and prolong life sufficiently for restoration. Valuable mechanical agents have by no means been discarded, but simply restrained by more discriminate reserve; and the expediency of employing them to be determined by due and careful estimate, in each individual case, of the comparative chances of their proving useful, or of shortening life by adding dangers of treatment to those of disease.

These conservative principles underlie and constitute the basis of modern therapeutics, and pervade every branch of practical medicine and surgery. By contrast with the past, the difference becomes strikingly apparent; and to say that these changes have been favorable to society, and amount to material and wonderful improvements, is but to utter the language of chemical experience and mortuary reports. The ratio of recoveries has been greatly increased, and the average duration of life prolonged, in proportion as these changes in practice have taken place.

This fragmentary sketch of progressive medicine expresses the sentiment and practice of the vanguard of the profession ; but this conservative practice has not yet been generally promulgated by the medical faculty, nor exemplified in the sick room by the mass of practitioners. Old Fogy still lags behind, content with his "lancet and pill-box," and unwilling to concede any eulogy to the Author of nature for his grand designs, or any skill in the protection of his own handiwork! The sluggard loiters in ignorance and content, but much the pity for that spot of society to which he assumes to dispense the benefits of medicine. These recent and wonderful evolutions in medical practice constitute a bright spot in the horizon of science and philanthropy, and reflect lustre upon the genius and enterprise of leading medical minds. The change is sound and logical as the laws of nature, and is destined to exemplify its truth and power in untold benefactions to society and suffering humanity.

Let us up and to the front, and so imbue our minds with the spirit of progressive medicine as to be ever capable of dispensing to society the utmost possible benefits of the noblest and most responsible of human professions.

[We must dissent from the general tone of the above article, in its tendency to the encouragement of expectancy. It is easy to pursue the routine of tonic and supporting treatment, but by it we lose the benefit of rational medicine. We like conservatism in all things, but when practiced unwarrantably, it becomes also an extreme, like that intended to be corrected. Forty years ago general blood-letting by some was perhaps practiced excessively, but we find much greater injury to suffering humanity in the opposite extreme now. There is no conservatism in discarding a valuable remedy. Unless violent acute inflammation be fought vigorously by sedative, counter irritative and depletory measures, destruction of vital organs may result. Such remedies may prove injurious when improperly used, but when applied with judgment and skill, are often indispensable in the treatment of disease. Diseases are different, and even opposite in their nature, and require different remedies accordingly. It is some times necessary to stimulate one organ and at the same time depress another. Cardiac sedation and cerebral stimulation are often demanded in acute inflammation. Homœopathic reliance on *vis medicatrix naturæ* is a very easy course to the practitioner. Much mental and physical labor is saved by it, at loss to the patient.—Editor.]

